# Implementation of an Anaesthesia Quality Improvement Programme to Reduce Fibreoptic Bronchoscope Repair Incidents

**DOI:** 10.1155/2020/1091239

**Published:** 2020-04-12

**Authors:** Hsiao-Feng Lu, Kuo-Chuan Hung, Min-Hsien Chiang, Johnson Chia-Shen Yang, Sheng-Dean Luo, Jo-Chi Chin, Chih-Hsien Wang, Cheuk-Kwan Sun, Shao-Chun Wu

**Affiliations:** ^1^Department of Anaesthesiology, Kaohsiung Chang Gung Memorial Hospital and Chang Gung University College of Medicine, Kaohsiung, Taiwan; ^2^Department of Anaesthesiology, Chi Mei Medical Center, Tainan, Taiwan; ^3^Division of Plastic and Reconstructive Surgery, Department of Surgery, Kaohsiung Chang Gung Memorial Hospital and Chang Gung University College of Medicine, Kaohsiung, Taiwan; ^4^Department of Otolaryngology, Kaohsiung Chang Gung Memorial Hospital and Chang Gung University College of Medicine, Kaohsiung, Taiwan; ^5^Department of Emergency Medicine, E-Da Hospital, School of Medicine for International Students, I-Shou University, Kaohsiung, Taiwan

## Abstract

**Background:**

This study was aimed at investigating the effectiveness of the implementation of a comprehensive quality improvement programme (QIP) for reducing the repair rate of the fibreoptic bronchoscope (FOB).

**Methods:**

A three-stage improvement strategy was implemented between January 2013 and December 2016. Stage one is the acquisition of information on violations of practice guidelines, repair rate, cost of repair, and incidence of unavailability of FOB during anaesthesia induction of the previous year through auditing. Stage two is the implementation of a quality improvement campaign (QIC) based on the results of stage one. Stage three is the programme perpetuation through monitoring compliance with policy on FOB use by regular internal audits. The effectiveness was retrospectively analyzed on a yearly basis.

**Results:**

The annual repair rate, repair cost, and incidence of FOB unavailability before the QIP implementation were 1%, 18,757 USD, and 1.4%, respectively. After QIC, the repair rate in 2013 dropped by 81% (from 1% in 2012 to 0.19% in 2013, *p* < 0.05). The annual repair cost fell by 32% from 18,758 USD (2012) to 12,820 USD (2013). Besides, the incidence of FOB unavailability plummeted by 71% from 1.4% to 0.4% during the same period. The annual repair rates and incidence of FOB unavailability remained lower in subsequent three years than those before QIP implementation.

**Conclusion:**

Implementation of a quality improvement programme was effective for reducing the rate and cost of FOB repair as well as unavailability rate, highlighting its beneficial impact on cost-effectiveness and patient safety in a tertiary referral center setting.

## 1. Introduction

A difficult airway is an emergency that can lead to life-threatening complications and medicolegal litigation [1]. Intubation using a fibreoptic bronchoscope (FOB), which consists of thousands of densely packed flexible glass fibres, is a cornerstone technique for managing both predicted and unpredicted difficult airways [2–4]. On the other hand, prolonged handling and inability to adhere to the standard practice guidelines, which are usually encountered in an emergency setting, may contribute to the damage of this delicate instrument. Furthermore, because of the wide discrepancy in diameter between the FOB and the tracheal tube, the tube may impinge on laryngeal structures on advancement [5], which may contribute to scope malfunction [6]. Improper handling of FOB during transportation, disinfection, cleaning, and sterilization has also been reported to increase the risk of FOB damage [6–10]. Because of the high costs of purchase, maintenance, and repair, some authors proposed to limit its use to the management of a nonemergent difficult airway [11].

Not only does FOB damage cause intubation failure that compromises patient safety but also the costly repair of FOB also accounts for the largest proportion of cost per use [11, 12]. The average repair cost had been reported to be up to 2959.44 USD per damage incident [11] or 17,891 EUR per year [6]. To address this issue, a number of studies have focused on the costs of fibreoptic tracheal intubation and the comparison of cost-effectiveness between the reusable FOB and single-use scopes without mentioning possible prevention strategies [6, 11–15]. Although two previous studies reported that implementation of a personnel training programme may decrease the repair rates and costs of bronchoscopy in a pulmonary care setting [16, 17], the impact of a quality improvement programme (QIP) on the repair rates and costs of FOB remains undetermined in an anaesthesia service setting. Therefore, this study was aimed at investigating the effectiveness of a three-stage QIP through identifying violations of standard practice guidelines, implementing a quality improvement campaign, and monitoring compliance with policy on FOB use.

## 2. Materials and Methods

### 2.1. Study Equipment, Design, and Setting

**Figure 1:**
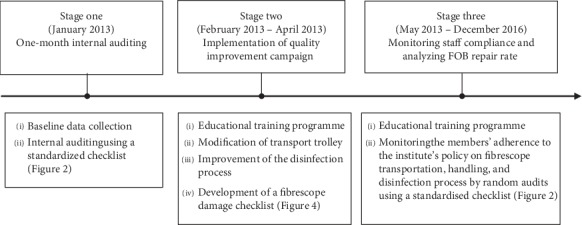
Schematic overview of a three-stage quality improvement programme for reducing fibreoptic bronchoscope (FOB) damage and cost of repair.

Between January 2013 and December 2016, a retrospective analysis of a three-stage QIP on FOB utilization and repair was performed at a tertiary referral center (Kaohsiung Chang Gung Memorial Hospital). Stage one (i.e., January 2013) of the programme involved an internal audit for the identification of the violations of practice guidelines, the repair rate, and the cost thus generated as well as the incidence of FOB unavailability during anaesthesia induction in the previous 12 months (i.e., January to December 2012). Five FOBs (Pentax Fibrescope FI-RBS, Pentax Medical Company, Montvale, NJ, USA), which were purchased before 2010, were available for use at the institute during the study period when no new FOB or other airway instruments (e.g., videolaryngoscopes) were available. Based on the data acquired, the second stage (stage two) of the programme included a three-month quality improvement campaign (February 2013 to April 2013) with strategies designed to reduce the FOB repair rate and cost as well as assessment of the effectiveness of the programme. Stage three of the QIP ensured successful continuation of the programme through regular internal audits every three months to monitor staff compliance and analyze the FOB repair rate. The manpower involved in the implementation of the QIP (e.g., internal audit, additional time involved in design, campaign, and monitoring) was financially supported by the institute without involving external contractors. A schematic presentation of the quality improvement programme is shown in Figure 1. The study was conducted in accordance with the Declaration of Helsinki and was approved by the Institutional Review Board (IRB) of Chang Gung Memorial Hospital (IRB No. 201800278B1). Informed written consent was waived because of the retrospective nature of the study.

### 2.2. Stage One: Auditing for Violations of Previous Standard Practice Guidelines, Unavailability Rate, Repair Rate, and Cost

**Figure 2:**
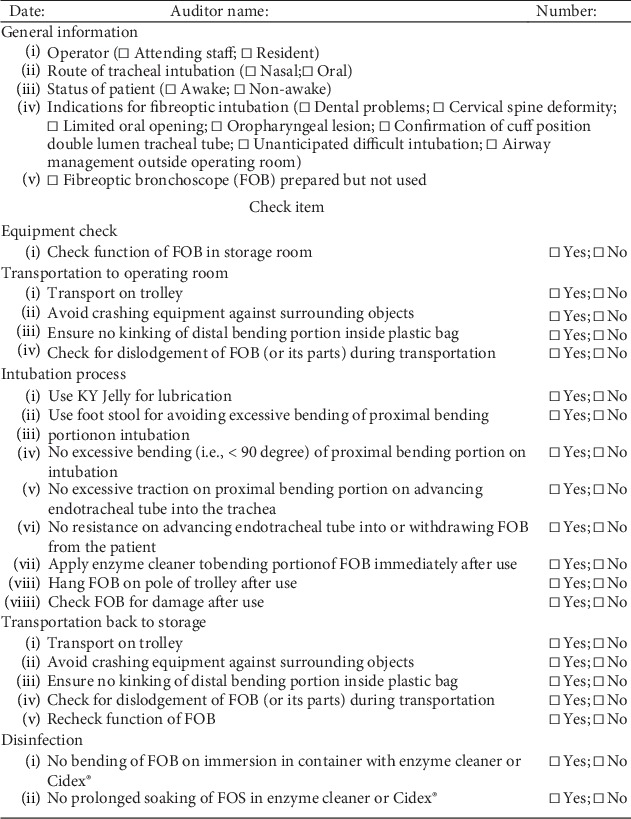
Internal audit checklist for monitoring compliance with standard practice guidelines on the proper use of the fibreoptic bronchoscope (FOB).

In view of the high cost of FOB repair in the year 2012, the compliance of the personnel of the Department of Anesthesiology with the established standard practice guidelines on FOB use at the institute was reviewed (Figure 2). A one-month internal auditing process including the identification of the violations of practice guidelines as well as unavailability rate, repair rate, and cost during the previous 12 months (January to December 2012) was conducted by an anaesthesia quality improvement team with members comprising three senior anesthesiologists and six nurse anesthetists with over ten years of clinical anaesthesia experience. A standard checklist with items covering the status of the patient (i.e., awake vs. nonawake) and indications for fibreoptic intubation as well as the whole process of FOB use (i.e., transportation, intubation, and disinfection) was adopted for the purpose of internal audit (Figure 2).

### 2.3. Stage Two: Quality Improvement Campaign

**Figure 3:**
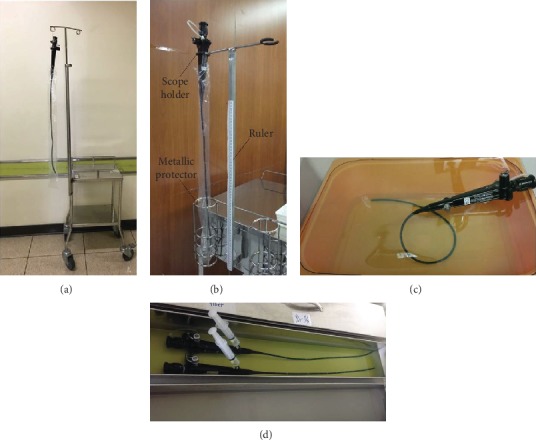
Modification of transport trolley and disinfection container designs for the protection of the fibreoptic bronchoscope (FOB). (a) Transport trolley before modification with high risk of scope damage during transportation. (b) Refined trolley design with installation of a scope holder and a metallic protector for the prevention of dislodgement of FOB from the pole and crashing of FOS against surrounding objects during transportation, respectively. A ruler extending through the length of the bending portion to ensure complete extension of FOB on hanging without kinking inside the plastic bag. (c) Bending during immersion in an enzyme cleaner or Cidex® in a container before improvement. (d) Use of a metal container of adequate length to allow FOB to extend to its full length without bending during immersion in the cleaning process as part of the quality improvement programme.

**Figure 4:**
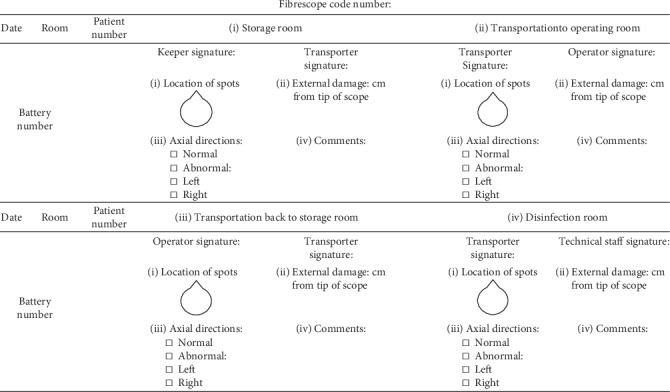
Fibreoptic bronchoscope (FOB) damage checklist for identifying the source and degree of damage after each use.

The campaign between February and April 2013 included the following: (1) an educational training programme focusing on proper FOB transportation and handling for all members of the anesthesiology department of the institute. An educational video was made to allow easy access. Residents who completed the training programme and showed reliable skills of FOB handling on manikins were allowed to perform fibreoptic intubation in patients under supervision. (2) To avoid dislodgement of FOB from the transport trolley and crashing of FOB against surrounding objects during transportation, the holding part of the pole was reinforced and swinging of the scope was prevented by placing the distal part into a metallic protector. A ruler extending through the length of the bending portion was put on the pole to ensure complete extension of FOB on hanging without kinking inside the plastic bag (Figures 3(a) and 3(b)). (3) The disinfection process is improved. For example, to prevent chemical damage of FOB from prolonged Cidex® sterilization that has been reported to be a significant contributor to scope damage [10], an alarm system with a timer set at 30 minutes was installed. In addition, a metal container of adequate length to allow FOB to extend to its full length without bending during immersion in an enzyme cleaner or Cidex® was used (Figures 3(c) and 3(d)). (4) A fibrescope damage checklist that had to be briefly completed and signed by the responsible personnel at each stage of use (i.e., storage, transportation, and disinfection) to help identify the source of damage (Figure 4) is developed. An internal audit was performed by the anaesthesia quality improvement team to ensure its correct completion. An internal audit was performed after the campaign to assess the rate of violation of the practice guideline. During the campaign, all members at the institute were not informed of the data regarding the rate of practice violation.

### 2.4. Stage Three: Auditing for Compliance and Constant Monitoring of Outcomes

Starting from May 2013, internal audits were performed every three months to monitor the members' adherence to the institute's policy on the FOB transportation, handling, and disinfection process by random audits using a standardised checklist (Figure 2). The repair rate and cost of FOB were recorded and analyzed.

### 2.5. Definitions and Outcome Measurement

Our institute, which did not sign a maintenance contract for FOB with the manufacturer, had to afford the cost of repair. According to the institute's policy, the FOB was sent for repair if the instrument was deemed unsuitable for assisting intubation after clinical evaluation by anesthesiologists. The repair rate was defined as the number of repairs as a percentage of the total number of patients receiving fibreoptic tracheal intubations performed by anesthesiologists regardless of units at the institute. An incidence of FOB unavailability was defined as one in which anesthetic induction could not be proceeded because of unavailability of FOB. The primary outcome was the reduction in the repair rate after implementation of the QIP, while the secondary outcome was the change in the incidence of FOB unavailability before and after the initiation of the programme.

To assess the efficacy of QIP, the baseline data from January 1, 2012, to December 31, 2012, were used for comparison. Information about the total number of procedures performed, the number of FOB repairs, repair costs, and the incidence of FOB unavailability during anesthetic induction was collected. The results were retrospectively analyzed on a yearly basis.

### 2.6. Statistical Analysis

The repair rate of bronchoscopy was reported to be 2.7% [16], of which about 12.7% to 69% of damage may be prevented [10, 16–18]. Assuming a preventable rate of 70% among all incidences of scope repair, the incidence of unavoidable damage is 30%. With this in mind, the goal of the present study was to keep an FOB repair rate close to 0.81% (i.e., 2.7 × 30%) which was the estimated rate of unavoidable FOB damage. This would require a sample size of 757 procedures in each group (80% power, two-sided test at a 5% level). Data are expressed as mean ± SD or number (%). Determination of the significance of difference in the repair rate and incidence of FOB unavailability during anesthetic induction between baseline and the periods after implementation of the QIP was based on the chi-square test or Fisher's exact test where appropriate. Other data such as repair costs were summarized using descriptive statistics. All data were entered into a database using Microsoft Excel (Microsoft Corp., Redmond, WA, USA), with statistical analysis performed with the SPSS 20.0 statistical software package (SPSS Inc., Chicago, IL, USA). All *p* values < 0.05 were considered statistically significant.

## 3. Results

### 3.1. Stage One: Identification of Violations of Standard FOB Practice Guidelines

The one-month internal audit on deviations from standard FOB practice guidelines of the personnel demonstrated that the majority of procedures were performed by attending staff (77.8%), the most common indication for FOB use was an anticipated difficult airway (57.3%), and the prevalent violation of standard practice guideline was intubation-related procedures (43.6%) (Table 1). There were 18 repair incidents during the baseline period (January to December 2012). The reasons for repair are bronchoscope bundle damage (77.8%), malfunction of lamps (11%), impaired angle control (5.6%), and worn external sheath (5.6%). The repair rate, repair cost, and incidence of FOB unavailability before the implementation of the QIP were 1%, 18,757 USD, and 1.4%, respectively (Table 2).

### 3.2. Stage Two: Implementation of the Quality Improvement Campaign

During the three-month intervention period, the education programme for the FOB transportation, handling, and disinfection process was provided to 121 members (anaesthetists, *n* = 23; residents, *n* = 6; anaesthesia nurse, *n* = 88; and disinfection technologists, *n* = 4). Following the campaign, the internal audit showed that the rates of violation of practice guidelines in the five aspects (Table 1) were all reduced to 0%.

### 3.3. Stage Three: Outcomes of the Quality Improvement Programme

The number of fibreoptic intubations, number of repairs, repair rate, repair costs, incidence of FOB unavailability before and after the implementation of the QIP are shown in Table 2. After completion of the quality improvement campaign, the repair rate in the year 2013 significantly dropped by 81% (from 1% in 2012 to 0.19% in 2013, *p* < 0.05). The annual repair cost fell from 18,758 USD in 2012 to 12,820 USD, representing a 32% reduction. Besides, the incidence of FOB unavailability plummeted by 71% from 1.4% to 0.4% during the same period. The long-term outcomes of the QIP are shown in Table 2. During the follow-up period of 3 years, the repair rates and incidence of FOB unavailability remained lower than those before QIP implementation.

## 4. Discussion

Although fibrescope-assisted tracheal intubation is indispensable in the management of a difficult airway in modern anaesthesia practice [2–4], there is a lack of literature addressing the impact of quality improvement programmes on FOB repair rate and cost in the anaesthesia service setting. The present study is the first to show that the repair rate and the associated cost as well as the incidence of unavailability could be significantly reduced through the implementation of a staged quality improvement programme including internal auditing to identify violations of practice guidelines, implementation of a multifaceted quality improvement campaign, and continuation of the programme through constant internal audits to ensure compliance of personnel with the institute's guidelines. These novel findings highlighted the cost-effectiveness of the implementation of a quality improvement programme on FOB use in the anaesthesia setting.

In the setting of pulmonary care, about 12.7% to 69% of all bronchoscope damage could be prevented [10, 16–18]. By the implementation of an educational programme to improve bronchoscope handling, it has been reported that the repair rate (i.e., 2.7% vs. 0.6%) and costs can be effectively reduced in bronchoscopy units [16]. That study also found that all episodes of equipment damage occurred during the procedure [16]. By contrast, instead of retrospectively tracing the causes of FOB damage, the present study examined the outcomes of a QIP which is a more proactive approach to identify the violations of practice guidelines that were kept to minimum through a quality improvement campaign and persistent quality maintenance through constant internal audits. The results of the current study revealed that although the majority of violations were intubation-related (43.6%), violations could happen at all stages of scope usage including transportation, equipment check, and disinfection (Table 1).

The repair rate, which is defined as the percentage of repair incidence out of all the procedures performed, was reported to range from 0.7% (i.e., 47/6654) [17] to 2.7% (i.e., 57/2074) [16] in the bronchoscopy setting. It has also been reported to range from 1.8% (i.e., 1/55) to 5.6% (i.e., 1/18) in the operating theater setting [11, 12]. The difference in the repair rate between the two settings may be attributed to the nature of the procedures being performed [6]. In the operating room (OR), the FOB was often used in patients with a difficult airway. Multiple intubation attempts and prolonged handling may increase the risk of FOB damage, and the equipment may be mishandled in emergent critical airway management. Before the implementation of the QIP, the repair rate was 1% at our institute, which is lower than that reported in previous studies (i.e., from 1.8% to 5.6%) [11, 12]. In contrast to the previous studies that included relatively small numbers of procedures (i.e., annual number of fibreoptic intubations (FOIs): 166 and 141, respectively), the current study investigated over 1500 intubations per year. Besides, most fibreoptic intubations were performed by experienced staff anesthetists (Table 1). These factors may contribute to the lower FOB repair rate compared to that in previous reports [11, 12]. Despite the relatively low repair rate, the results of the current study demonstrated that further improvement could be achieved through a well-planned QIP.

Our audit report showed that the frequency of an unanticipated difficult airway requiring FOB for management was up to 1.8% (Table 1), further highlighting the adverse impact of FOB unavailability on patient safety. Unavailability of FOB can also result in prolonged patient waiting time, reducing the efficacy of operating room utilization. Therefore, the improvement in FOB unavailability from 1.4% to 0.4% after QIP implementation in the present study may signify more effective use of operating room resources.

The present study is the first to demonstrate the long-term benefit of QIP in reducing the repair rate as reflected in the persistent improvement up to three years after QIP implementation (Table 2). Besides, the drop of the total repair cost by 32%, 100%, 55%, and 57% after the implementation of the QIP in the years 2013, 2014, 2015, and 2016, respectively, compared to that in 2012 further supports the cost-effectiveness of the programme. It is interesting that although the annual repair cost was reduced, the average cost per instance of repair was still higher in the following years (i.e., 2015 and 2016) than before the implementation of the QIP (i.e., 2012) (Table 2). We suggested that because of the high procedure volume and repeated use of FOB, the scope of repair may begin with replacement of relatively minor components during the baseline period (i.e., 2012) to complete refurbishment in the following years, leading to the relatively high average cost per instance of repair in the following years.

In view of previous episodes of FOB damage from falling, crashing with surrounding objects, and kinking, the transport trolley in the operating room was redesigned to allow firm grasp and complete extension of the scope which can be kept inside a metallic protector to avoid swinging during transportation. On the other hand, the risk of FOB damage during transportation remains high when the equipment is used outside the operation theater where the trolley is not available. The risk is further elevated by the relatively short response time of only five minutes according to the institute's policy. In addition, the difficulty in airway management in the nonoperating theater setting has been reported to be high because of the need for immediate response to unfamiliar patients who are usually hypoxic or hemodynamically unstable in an environment without adequate resuscitation equipment [19]. Another study also discouraged the use of FOB in an emergency setting to minimize the risk of damage [11].

A fibrescope damage checklist to identify the source of damage at different stages of FOB use (Figure 4) may help minimize damage through improving a particular procedure at risk. Besides, awareness of preexisting scope disorders could alert the operator to the risk of further damage to the instrument, thereby encouraging strict adherence to the handling principle. As FOB is usually used for the management of an unanticipated difficult airway in emergency conditions, keeping track of the performance status of FOB could minimize unexpected malfunction and enhance patient safety.

There are several limitations in the current study. First, the effect of availability of other airway instruments (e.g., videolaryngoscopes) on decreasing the number of fibreoptic intubations and reducing the repair costs was not evaluated. Second, the operator's experience in difficult airway manipulation, which may affect the probability of scope mishandling, was not assessed. Third, the impact of FOB unavailability on the outcomes of airway management was not analyzed. Fourth, because the implementation of the QIP did involve costs of manpower and refining devices, additional costs were inevitable. However, since all the costs of manpower were incorporated into the salaries of the hospital employees who participated in the project, the overall cost could not be assessed. Besides, we did not keep a precise record of the time required for QIP implementation for evaluating the cost of the time involved. Regarding the devices used for the QIP such as the transport trolley and containers of adequate length, they were mere modifications of existing products by our Department of Repair and Maintenance and were not involved in the purchase of expensive materials. Therefore, the costs were minimal. Finally, the exact causes of scope damage in the year 2012 remain unclear during the internal audit (stage one). Nevertheless, the incidence of scope damage and cost of repair were significantly reduced through the implementation of a QIP to ensure personnel compliance with the practice guidelines.

In conclusion, the results of the present study showed that a staged quality improvement programme not only could offer long-term improvement in the cost-effectiveness of FOB use but also could reduce the incidence of scope damage and improve the quality of patient care. A similar strategy for quality improvement may be adopted by other medical institutes. Nevertheless, further studies are required to evaluate the impact of such programmes on patient safety.

## Figures and Tables

**Table 1:** Results of one-month internal audit regarding indications for fibreoptic bronchoscope (FOB) utilization, operator's experience, and violations of the standard operating procedure (*n* = 117).

Items	*N* (%)
Intubation performed by residents	26 (22.2)
Indications for FOB application	
Anticipated difficult airway	67 (57.3)
Confirmed position of a double-lumen tube	21 (17.9)
Unanticipated difficult airway	2 (1.8)
Others (e.g., to avoid tooth injury)	27 (23.1)
Violations of standard practice guidelines	
Unnecessary transportation^∗^	11 (9.4)
Equipment check-related procedures	39 (33.3)
Intubation-related procedures	51 (43.6)
Transportation-related procedures	42 (35.9)
Disinfection-related procedures	19 (16.2)

^∗^Transportation of FOB to the operating room without being used.

**Table 2:** Changes in parameters related to fibreoptic bronchoscope (FOB) use, repair, cost, and unavailability before and after the implementation of the quality improvement campaign (QIC).

Variables	Pre-QIC (2012)	Year of QIC (2013)	Follow-up		
			Post-QIC Year 1 (2014)	Post-QIC Year 2 (2015)	Post-QIC Year 3 (2016)
Number of tracheal intubations	23,451	24,093	24,067	28,468	26,327
Number of FOB	5	5	5	5	5
Number of FOI	1800	1598	1662	1596	1661
Number of repairs	18	3	0	3	3
Repair rate^∗^	1%	0.19%^‡^	0^‡^	0.19%^‡^	0.18%^‡^
Mean FOI per FOB	360	320	332	319	332
Total repair costs (USD)	18,758	12,820	0	8470	8137
Drop in total repair costs compared with the baseline	—	32%	100%	55%	57%
Cost of repairs per procedure (USD)	10.42	8.02	0	5.31	4.90
Average cost per instance of repair (USD)	1042	4273	0	2823	2712
Frequency of FOB unavailability	1.4%	0.4%^‡^	0.3%^‡^	0.3%^‡^	0.3%^‡^

FOB: fibreoptic bronchoscope; FOI: fibreoptic intubation; USD: US dollars. ^∗^(Number of repairs/total number of procedures performed in the same year) × 100%. ^‡^*p* < 0.05 compared with the pre-QIC period.

## Data Availability

The data used to support the findings of this study are included within the article.
